# Whole-Genome Shotgun Sequencing of the First Observation of *Neisseria meningitidis* Sequence Type 6928 in India

**DOI:** 10.1128/genomeA.01232-16

**Published:** 2016-11-03

**Authors:** Balaji Veeraraghavan, Ayyan Raj Neeravi, Naveen Kumar Devanga Ragupathi, Francis Yesurajan Inbanathan, Agila Kumari Pragasam, Valsan Philip Verghese

**Affiliations:** a Department of Clinical Microbiology, Christian Medical College, Vellore, Tamil Nadu, India; b Department of Child Health, Christian Medical College, Vellore, Tamil Nadu, India

## Abstract

*Neisseria meningitidis* is one of the leading global causes of bacterial meningitis. Here, we discuss the draft genome sequences of two *N. meningitidis* strains, isolated from bloodstream infections in two pediatric patients at a tertiary care hospital in South India. The sequence data indicate that strains VB13856 and VB15548 encode genomes of ~2.09 Mb in size with no plasmids.

## GENOME ANNOUNCEMENT

*Neisseria meningitidis* is a known cause of bacterial meningitis. Serogroup A meningococcal epidemics are known to be responsible for high morbidity and mortality, particularly in developing countries (1). Similarly, there is an indication of evolution in meningococcal disease due to increased incidence of serogroups C, Y, and W-135 in countries like India (2). The worldwide epidemiology of invasive meningococcal disease varies markedly by region and over time.

In this study we present the draft genome sequences of two *N. meningitidis* strains (VB13856 and VB15548), isolated from bloodstream infections in two different pediatric patients who are natives of South India. The isolates were identified to be serotype C with respective antisera. VB13856 was observed to be susceptible to penicillin, while VB15548 was moderately susceptible. Both isolates were observed to be susceptible to cefotaxime but resistant to ciprofloxacin. To further understand the molecular makeup of the isolates, we determined their whole genome sequences. Whole-genome shotgun sequencing was performed using Ion Torrent PGM with 400-bp chemistry. The data were assembled *de novo* using AssemblerSPAdes version 5.0.0.0 embedded in Torrent suite server version 5.0.3. Assembly of the raw reads of the isolates VB13856 and VB15548 showed 137 and 145 contigs (≥500 bp), with 100× and 83× coverage, respectively. The sequence annotation was performed in PATRIC, the bacterial bioinformatics database and analysis resource (http://www.patricbrc.org) (3), the Rapid Annotation using Subsystem Technology (RAST) pipeline (http://rast.nmpdr.org) (4, 5), and the NCBI Prokaryotic Genome Automatic Annotation Pipeline (PGAAP, http://www.ncbi.nlm.nih.gov/genome/annotation_prok). The genome of VB13856 was identified to be 2,120,762 bp with 2,634 coding sequences (CDSs), 3 rRNAs, and 44 tRNAs. The annotation revealed one ARDB and 16 CARD antimicrobial resistance genes. In addition, 33 and 75 virulence factors were identified by the VFDB and Victors databases, respectively (https://www.patricbrc.org). Similarly for VB15548, 2,127,664 bp containing 2,643 CDSs, three rRNAs, and 45 tRNAs were identified, along with one ARDB and 17 CARD antimicrobial resistance genes, and 32 and 75 virulence factors identified by the VFDB and Victors databases, respectively.

Next-generation sequencing also revealed that both strains were of sequence type (ST) 6928, as analyzed by the MLST version 1.8 tool (https://cge.cbs.dtu.dk//services/MLST/) (6). To the best of our knowledge, this is the first report of ST6928 *N. meningitidis* strains in India. ResFinder version 2.1 and PlasmidFinder version 1.3 (http://www.cbs.dtu.dk/services) returned no known genes in both the isolates. However, the phenotypic resistance to ciprofloxacin could be explained by the presence of RND and *mac*A efflux genes.

Identification of *N. meningitidis* ST6928 in India necessitates the continuous monitoring of such clinical cases. Though genes corresponding to ciprofloxacin resistance were not identified, further studies are required to study the exact resistance and virulence mechanism to better understand and control the spread of bacterial meningitis disease.

### Accession number(s).

Whole-genome shotgun sequences of isolates VB13856 and VB15548 were submitted to NCBI under the accession numbers MDSH00000000 and MDSI00000000, respectively. The versions described in this manuscript are the first versions, MDSH01000000 and MDSI01000000.
